# Hilly neighborhoods are associated with increased risk of weight gain among older adults in rural Japan: a 3-years follow-up study

**DOI:** 10.1186/s12942-019-0174-z

**Published:** 2019-05-10

**Authors:** Kenta Okuyama, Takafumi Abe, Tsuyoshi Hamano, Miwako Takeda, Kristina Sundquist, Jan Sundquist, Toru Nabika

**Affiliations:** 1 Center for Community-based Healthcare Research and Education (CoHRE), Organization for Research and Academic Information, Shimane University, 223-8 Enya-cho, Izumo-shi, Shimane 693-8501 Japan; 2 Department of Sports Sociology and Health Sciences, Faculty of Sociology, Kyoto Sangyo University, Motoyama, Kamigamo Kita-ku, Kyoto, 603-8555 Japan; 3 Center for Primary Health Care Research, Clinical Research Centre (CRC), Skåne University Hospital, Lund University, Building 28, Floor 11, Jan Waldenströms Gata 35, 205 02 Malmö, Sweden; 4 Department of Functional Pathology, Faculty of Medicine, Shimane University, 89-1, Enya-cho, Izumo-shi, Shimane 693-8501 Japan

**Keywords:** Neighborhood, Rural, Slope, Weight change, Older adults

## Abstract

**Background:**

Neighborhood environments have been regularly associated with the weight status. Although the evidence is mostly limited to adults residing in western urban settings, the weight status of older adults living in rural areas is also assumed to be significantly affected by their neighborhood environments. This study aimed to identify environmental attributes specific to rural areas that could affect the risk of longitudinal weight gain among older adults (≥ 65 years) in Japan.

**Methods:**

We examined five environmental attributes, i.e., land slope, public transportation accessibility, residential density, intersection density, and the availability of parks and recreational centers, measured by the geographic information system. Our analysis was based on 714 subjects participated in Shimane Community-based Healthcare Research and Education study in 2012 and 2015. Multinomial logistic regression model was conducted to examine the association between each neighborhood environmental attribute and weight change status (gain, loss and unchanged).

**Results:**

We observed a significant increase in the risk of weight gain as the steepness of the neighborhood land slope increased. There was no significant association between other environmental attributes and risk of weight gain as well as weight loss among older adults.

**Conclusion:**

Living in hilly neighborhoods was associated with increased risk of weight gain among rural Japanese older adults. Future research should consider region-specific environmental attributes when investigating their effect on older adults’ weight status.

## Background

Weight change among older adults increases their mortality risk [1]. This has been established in several longitudinal studies [2, 3]. The burdens of excess weight were estimated for causing 3.4 million deaths globally [4], and significant increase of medical cost burden attributed to excess weight has been observed in the US and east Asian countries, including Japan with the consistent rise of obesity prevalence [5–7]. Neighborhood environments are known to have substantial impacts on the health and functioning of older adults, and also weight change [8]. While several studies [9–11] have longitudinally assessed the change in weight-related measures and neighborhood environments, one of them [9] have found significant associations between the neighborhood environment and weight status among older adults. These studies were mostly conducted in western urban settings, and the evaluation of neighborhood environments is primarily based on urban environmental attributes such as residential density, land use mix, street connectivity, and retail floor ratio, measured by geographic information system (GIS) [12]. These urban environmental measures are not thought to be associated with the health behaviors of the rural population [13, 14] and are rarely applicable in the assessment of rural neighborhood environments [15].

Rural residents are at a higher risk of chronic health conditions such as cardiovascular disease and some forms of cancer, as well as modifiable risk factors such as physical inactivity and obesity when compared to those of urban and suburban counterparts [16, 17]. For example, lower residential density and population density have been consistently found to be associated with physical inactivity internationally [18–20]. Furthermore, a higher percentage of aging population is more often observed in rural settings across the world [21]. Despite these, there is a lack of studies in rural settings, especially among elderly people.

Japan is defined as a “super-aged society,” a term used to describe a society where more than 21% of the total population is aged above 65 years according to the standard definition by the United Nations and World Health Organization. As of 2015, the aging rate reached an unprecedented level of 26% in Japan and was the highest in the world [22]. Although there are a few studies in Japan which have examined neighborhood environmental characteristics, weight status, and other health-related outcomes among older adults using GIS [23–27], those are limited in urban settings. In those studies, some environmental attributes, such as access to public transit, elevation, and land slope were found to be associated with physical activity, hypertension, and diabetes [25–27]. There is a growing interest to measure not only built environment but also natural environment as those are thought to have significant impact on population health, especially in rural settings where urban attributes are not applicable [28]. Slope land is a unique geographic feature of Japan and the cause for hilly neighborhoods [29]. Identifying environmental attributes in rural settings is critical to enact policy and environmental level intervention in order to improve the health of vulnerable population. From these backgrounds, the purpose of this study is to identify environmental attributes specific to vulnerable areas, i.e., rural areas, that can affect weight change among Japanese older adults by longitudinal study design.

## Methods

### Data

This study used longitudinal data of older adults (≥ 65 years) who participated in our Shimane CoHRE study. Shimane CoHRE is a cohort study in collaboration with three municipalities in the Shimane Prefecture to identify potential factors of healthy aging among rural residents by investigating their lifestyle habits and any chronic conditions they may have had. Shimane prefecture is a rural mountainous region located in western Japan. In Japan, urban area refers to the area with basic unit census block with population density 4000 or more per square km, and whose total population exceeds 5000. Rural-mountainous areas defined as the areas whose 50–80% or more of the land is covered by the forest, and cultivated lands are below 10% of total land areas and are mostly hilly [30]. Shimane has the third highest aging rate among the 47 prefectures in Japan (Total population in Shimane: 694 352; Japan: 12 483 901, the percentage of the population aged 65 years and above in Shimane: 32.5%; Japan: 26.6%, population density: 103.5 person/km^2^; Japan: 340.8 person/km^2^) [31]. The current study utilized the CoHRE data for 2012 (baseline) and 2015 (follow-up). The study subjects were those who participated in the annual health checkup, and agreed to participate in Shimane CoHRE study. Among 2147 people, 1630 subjects (≥ 65 years) participated in the baseline study. Among them, 926 subjects participated in the follow-up survey in 2015, of which, 212 subjects were excluded since data for relevant variables were missing. In the final study, there were 714 subjects. We examined whether there are differences in terms of basic characteristics, i.e., gender, age, and baseline weight, between the subjects included and not included in the study. There was no significant difference in terms of gender and baseline weight (*p* = 0.081, *p* = 0.158, respectively). Although there was statistically significant difference in terms of age (*p* < 0.001), the difference in median age value was only 1.0 (those included: 70.0, those not included: 71.0). All subjects have not moved their residential location over 3 years. The study protocol and procedure used to secure the informed consent of the participants were approved by the Ethics Committee of Shimane University #2888.

### Outcome variable

The weight of each participant was measured objectively by trained healthcare practitioners. These data were used as primary outcomes. The weight change ≥ 2.5 kg over 3 years was used as the cutoff point for weight gain and loss statuses in accordance with previous studies [2, 3, 32].

### Exposure variable

We used a total of five environmental attributes, i.e., land slope, public transportation accessibility, residential density, intersection density, and park and recreation center availability, within 400 m and 800 m network buffers from each residential point of the subjects in the study. We set multiple buffers of 400 and 800 m based on actual street network data, which are commonly defined as appropriate activity spaces for people in neighborhood studies [12]. The mean land slope, which expresses the steepness of hilly neighborhoods [23, 27], was computed based on the elevation and degree of Slope 5th Mesh Data (as of 2011). We computed the mean land slope within network buffers by calculating the value of slope stored in each 50 m grid which intersected with network buffers. Hilliness was represented by an angular unit: degree. These data were obtained from the National Land Numerical Information (NLNI), which is publicly available GIS data administered by the National Land Information Division, National Spatial Planning, and Regional Policy Bureau of Japan. Further, accessibility of public transportation, was computed using the number of bus stops and railway stations within each network buffer based on the bus stop and railway station point data as of 2010, available from NLNI. The residential density was computed using the number of households within each network buffer based on the data of the basic block unit households (as of 2010), which is the smallest Japanese census statistics area from the ArcGIS data collection by Esri Corporation, Tokyo, Japan (Esri Japan). The value of number of households stored in point data were summed which fallen within network buffers, and was used as residential density. Intersection density was computed as street connectivity using the number of intersections with three or more legs within each network buffer, based on the road network data collected by Esri Japan for 2012. The availability of park and recreational centers was computed within each network buffer based on the data on city parks as of 2011 and cultural facilities as of 2012, both obtained as point data from NLNI. All spatial analyses were conducted using ArcGIS 10.0 (Esri Inc., Redland, CA).

### Covariates

In the analysis, age (continuous), gender (male vs. female), baseline body weight (continuous), smoking habits (smoker vs. nonsmoker), drinking habits (drinker vs. non-drinker), having a driving license (yes vs. no), and educational attainment were included as covariates. All of this information was collected using self-reported questionnaires and face-to-face interviews. Data on educational attainment were collected through face-to-face interviews by talking to the participants about the duration of their education, and 12 years (high school graduate and above) was used as the cutoff.

### Statistical analysis

Descriptive statistics showed the characteristics of all study subjects. Summary statistics and correlation analysis were conducted for five neighborhood environment variables. Multinomial logistic regression was conducted to predict that the odds of weight gain or weight loss, when compared to weight change status, would remain unchanged over 3 years. The exposure variables, namely, the neighborhood environment variables, were all included separately in the model with both continuous and categorical measures. The mean land slope, residential density, and intersection density were included in the analysis as a quartile categorical value. The public transit stop density and park and recreational center were included in the analysis as a binary categorical value (more than one versus none) since these measures were absent in the neighborhoods of many subjects. To avoid multicollinearity among exposure variables, each variable was included separately in the model by adjusting for all covariates. All statistical analyses were conducted using R 3.4.3.

## Results

Basic characteristics of study subjects are shown in Table 1. The weights of 72.0% of the people did not change by more than 2.5 kg over 3 years, while 9.2% of the people gained more than 2.5 kg, and 18.8% of people lost more than 2.5 kg. The median age was similar across people whose weight status remained unchanged [70.0 years, interquartile range (IQR) = 68.0, 71.0], gained (70.0 years, IQR = 67.0, 73.0), and lost (71.0 years, IQR = 68.0, 71.0). The median weight at the baseline was also similar across all groups. Around one-third of the participants had more than 12 years of education across all groups. More than 75% of participants had driving licenses across all groups.

**Table 1:** Characteristics of the participants by weight change status

	Weight change status (≥ 2.5 kg) for 3 years		
	Unchanged	Gained	Lost
n (%)	514 (72.0)	66 (9.2)	134 (18.8)
Age [median (IQR)]	70.0 (68.0, 71.0)	70.0 (67.0, 73.0)	71.0 (68.0, 71.0)
Gender: male/female (%)	191/323 (37.2/62.8)	33/33 (50.0/50.0)	45/89 (33.6/66.4)
Weight [median (IQR)]	52.25 (46.4, 57.9)	54.80 (48.1, 60.2)	53.75 (49.8, 59.6)
Smoking: non-smoker/smoker (%)	485/29 (94.4/5.6)	50/16 (75.8/24.2)	129/5 (96.3/3.7)
Drinking: non-drinker/drinker (%)	245/269 (47.7/52.3)	38/28 (57.6/42.4)	57/77 (42.5/57.5)
Education: ≥ 12 years/< 12 years (%)	159/355 (30.9/69.1)	21/45 (31.8/68.2)	33/101 (24.6/75.4)
Driving license: no/yes (%)	117/397 (22.8/77.2)	15/51 (22.7/77.3)	32/102 (23.9/76.1)

Table 2 summarizes the five neighborhood environment variables and their correlations for 400 m and 800 m network buffers. The land slope was negatively associated with all other environment variables. Residential density and intersection density were highly correlated in both 400 m and 800 m network buffers (r = 0.75, and r = 0.86, respectively).

**Table 2:** Summary of five neighborhood environment variables and their correlation coefficient

N	714	714	1	2	3	4	5
	Mean (SD)	Median (IQR)					
*400* *m network buffer*							
1. Land slope	9.62 (4.66)	8.87 (5.82, 12.64)	1.00				
2. Public transit stop density	1.16 (1.57)	1.00 (0.00, 2.00)	− 0.33	1.00			
3. Residential density	111.77 (134.59)	64.00 (26.00, 142.00)	− 0.58	0.64	1.00		
4. Intersection density	5.37 (5.60)	4.00 (2.00, 7.00)	− 0.57	0.63	0.75	1.00	
5. Park and recreational center	0.22 (0.80)	0.00 (0.00, 0.00)	− 0.24	0.19	0.27	0.33	1.00
*800* *m Network buffer*							
1. Land slope	10.77 (4.75)	10.08 (6.63, 14.52)	1.00				
2. Public transit stop density	2.37 (2.59)	2.00 (1.00, 3.00)	− 0.23	1.00			
3. Residential density	209.11 (232.70)	126.00 (47.25, 274.00)	− 0.60	0.62	1.00		
4. Intersection density	12.13 (11.76)	9.00 (4.00, 16.75)	− 0.63	0.64	0.86	1.00	
5. Park and recreational center	0.70 (1.42)	0.00 (0.00, 1.00)	− 0.35	0.37	0.55	0.60	1.00

**Fig. 1:**
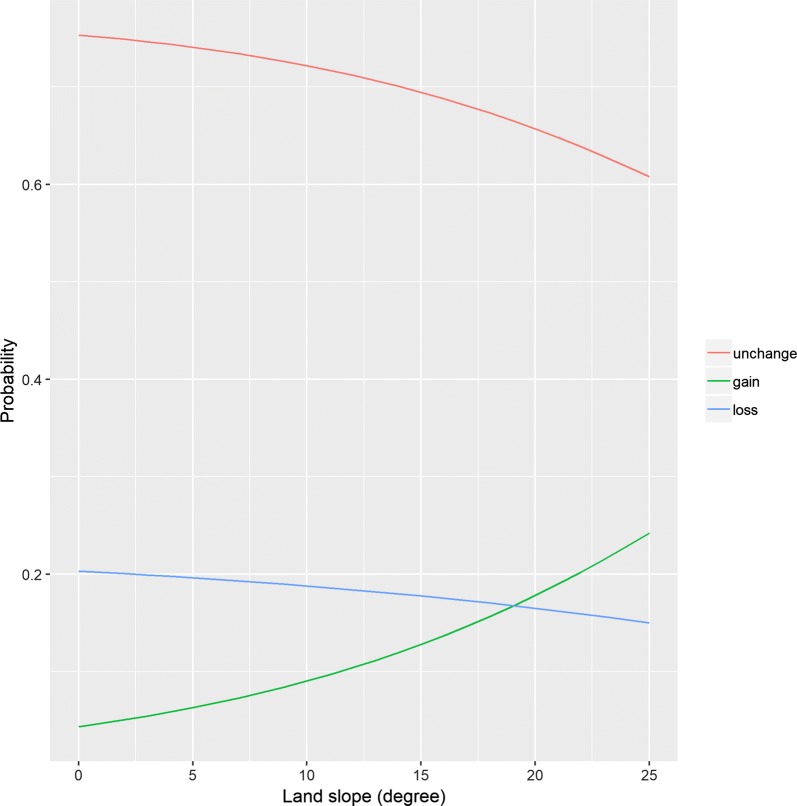
Predicted probabilities of weight change by land slope in 400 m network buffer

**Fig. 2:**
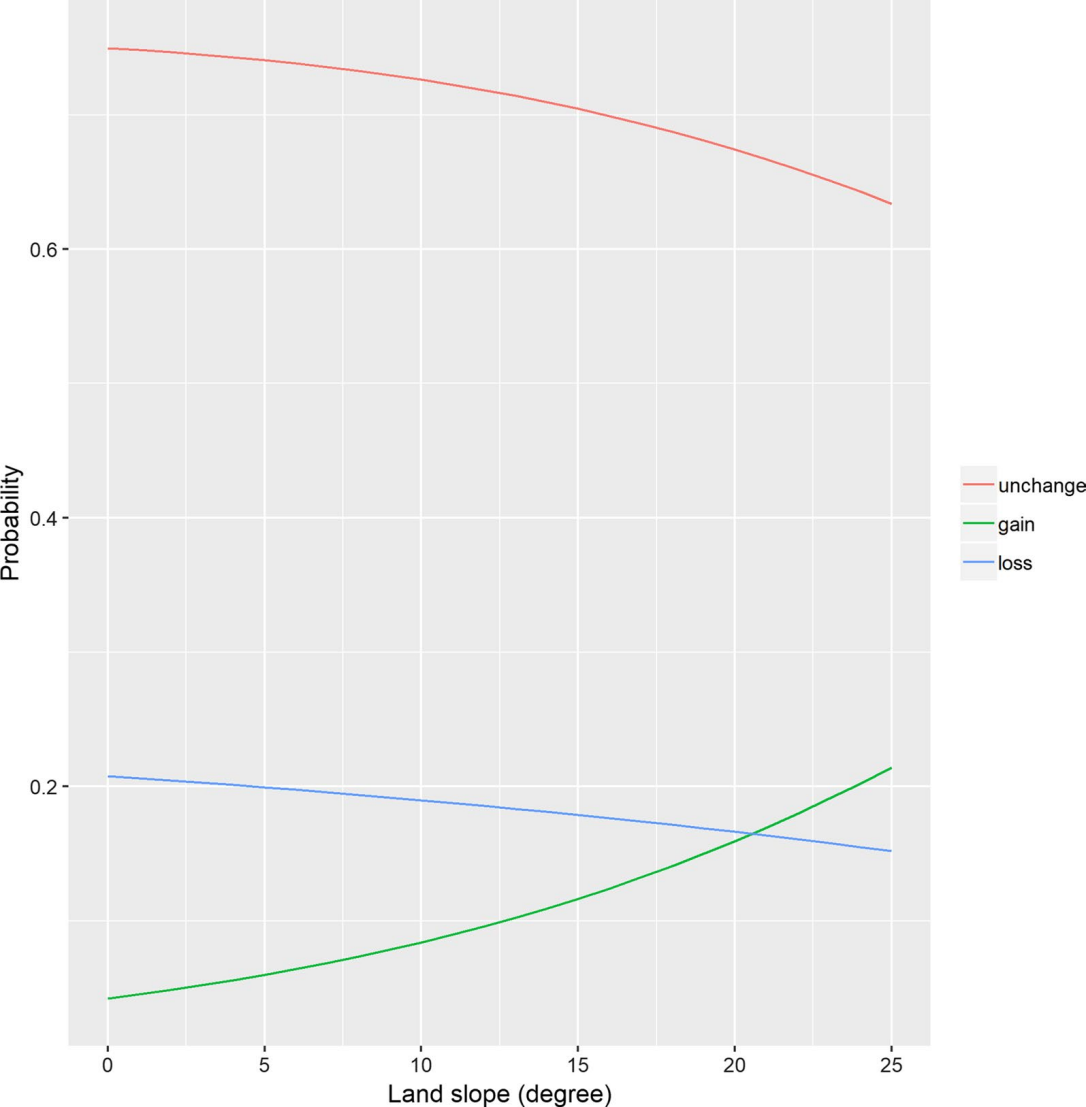
Predicted probabilities of weight change by land slope in 800 m network buffer

Table 3 presents the results of the multinomial logistic regression analysis for weight change status. The model included the neighborhood environment variables as categorical measures showed that land slope was significantly associated with weight gain for both 400 m and 800 m network buffers. While there was no significant increase in the odds of weight gain in the second quartile, taking the first quartile as a reference, there was a significant increase in the odds of weight gain in the third and fourth quartiles [Odds ratio (OR) 2.351, (95% Confidence interval (CI) 1.048, 5.275); OR 2.578, (95% CI 1.175, 5.654)] for the 400 m network buffer. The result indicates that living in the area with third or fourth quartile of slope had 2.4 or 2.6 times higher odds of weight gain. For the 800 m network buffer, there was a significant increase in the odds of weight gain in the second, third, and fourth quartiles when compared to the first quartile [OR 3.013, (95% CI 1.207, 7.521); OR 3.399, (95% CI 1.404, 8.229), OR 3.084, (95% CI 1.295, 7.343)]. No significant association was observed between weight loss and land slope. There was a significant decrease of odds in the third quartile of residential density within the 400 and 800 m network buffer when compared to the first quartile [OR 0.414, (95% CI 0.190, 0.899), OR 0.417, (95% CI 0.197, 0.880), respectively]. Public transit stop density, intersection density, and the availability of parks and recreational centers were not significantly associated with either weight gain or loss for both the 400 and 800 m network buffers. The model, including neighborhood environment variables as the continuous measure, showed that the odds of weight gain were positively associated with land slope (results not shown). Figure 1 shows the predicted probabilities of weight change with neighborhood land slope within 400 m network buffer. It shows that probabilities of weight gain increases as degree of land slope increases. When the network buffers were extended to 800 m, the significant association between weight gain and land slope was still observed, and similar trend was observed (Fig. 2). No significant association was observed between weight loss and land slope for both 400 m and 800 m network buffers. Other neighborhood environment variables were not associated with either weight gain or loss for both 400 m and 800 m network buffers.

**Table 3:** Multinomial logistic regression for the association between weight status and each neighborhood environmental attributes

Neighborhood environmental attributes	400 m Network buffer				800 m Network buffer			
	Weight gain		Weight loss		Weight gain		Weight loss	
	OR	95% CI	OR	95% CI	OR	95% CI	OR	95% CI
Land slope (2nd vs. 1st)	1.295	(0.527, 3.184)	0.798	(0.460, 1.385)	*3.013*	*(1.207, 7.521)*	1.223	(0.712, 2.101)
Land slope (3rd vs. 1st)	*2.351*	*(1.048, 5.275)*	1.007	(0.591, 1.716)	*3.399*	*(1.404, 8.229)*	1.069	(0.617, 1.852)
Land slope (4th vs. 1st)	*2.578*	*(1.175, 5.654)*	0.837	(0.480, 1.461)	*3.084*	*(1.295, 7.343)*	0.968	(0.561, 1.671)
Public transit stop density (≥ 1 vs. 0)	0.607	(0.356, 1.035)	0.851	(0.574, 1.261)	0.847	(0.466, 1.538)	1.151	(0.716, 1.848)
Residential density (2nd vs. 1st)	0.566	(0.270, 1.189)	0.830	(0.487, 1.416)	0.602	(0.292, 1.239)	1.389	(0.822, 2.347)
Residential density (3rd vs. 1st)	*0.414*	*(0.190, 0.899)*	0.622	(0.363, 1.065)	*0.417*	*(0.197, 0.880)*	0.682	(0.387, 1.202)
Residential density (4th vs. 1st)	0.952	(0.473, 1.913)	0.912	(0.513, 1.618)	0.803	(0.390, 1.654)	1.125	(0.630, 2.009)
Intersection density (2nd vs. 1st)	0.491	(0.221, 1.092)	1.433	(0.854, 2.406)	0.834	(0.418, 1.663)	0.850	(0.496, 1.454)
Intersection density (3rd vs. 1st)	0.725	(0.349, 1.508)	1.302	(0.754, 2.248)	0.568	(0.260, 1.239)	1.152	(0.686, 1.936)
Intersection density (4th vs. 1st)	0.660	(0.323, 1.349)	1.112	(0.646, 1.915)	0.835	(0.408, 1.711)	0.851	(0.484, 1.495)
Park and recreational center (≥ 1 vs. 0)	0.529	(0.179, 1.557)	1.081	(0.569, 2.052)	1.432	(0.803, 2.554)	1.045	(0.671, 1.630)

Each neighborhood environmental attribute was included separately

Age, gender, baseline weight, smoking, drinking, education attainment, and driving license were adjusted

Italic shows significance *p* < 0.05

## Discussion

This study sought to identify the effect of environmental attributes specific to rural areas on the risk of longitudinal weight change status among older adults in Japan. This was the first longitudinal study that analyzed the impact of the GIS measured neighborhood environment on the risk of weight change status among older adults in rural areas of Japan. The results of this study showed that hilly neighborhoods were associated with an increased risk of weight gain. The finding was inconsistent with other recent studies which examined the effects of hilly neighborhoods on the risk of diabetes in a cross-sectional fashion [27].

The previous study reported the preventive effect of living in hilly environments for poorly controlled diabetes mellitus among older adults [OR 0.82, (95% CI 0.70, 0.97)] [27]. The study explained that unconscious training for physical function by walking around the hilly neighborhood was a possible reason for the preventive effect. However, an increase in the degree of steepness may become a barrier for older adults to go out and walk around in their neighborhoods. In fact, the study area of that previous study was not as hilly as our study setting (mean land slope: 3.03 vs. 9.62). Furthermore, one study found a negative association between land slope and the likelihood of engagement in sports among older adults, i.e. living in hilly neighborhood was associated with lower likelihood of sports activity [23]. As we did not analyze to study this possible causal mechanism, further research is necessary, mainly using objectively measured data on walking time and physical activity, as well as data on the extent to which older adults go out of the house. The role of this information in mediation must be examined to understand this mechanism.

It was found that almost all other environmental attributes were not significantly associated with weight change status among older adults. While access to public transit was increasingly reported as an important neighborhood environmental attribute for health status in both urban and rural settings [26, 33], we did not find an association in our study. This may be because the public transit system should serve people to the extent that they could rely on it for their daily errands, such as commuting, shopping and accessing healthcare. However, public transit does not seem to serve older adults well, based on public transit stop density measures concerning the participants in this study (median, i.e., 400 m = 1; median, i.e., 800 m = 2). Also, the lack of information on whether the participants utilized public transit limited our analysis and, as a result, this study does not reveal the effect of access of public transit on the health of older adults. Residential density (except for the significant negative association of weight gain between the third and first quartiles), intersection density, and the availability of park and recreational centers, which are commonly known as critical environmental attributes for older adults’ health in urban settings, were not associated with weight change status in this study. A possible explanation for each attribute is that for residential density, the significant decrease in odds observed between the third and first quartiles is consistent with previous findings [15]. However, as this was only observed within when residential density was categorized, and no dose–response relationship was detected, the finding might have been a result of the sample distribution or arbitrary set neighborhood space. In addition, residential density is a measure of the degree as to how a neighborhood is occupied by residents, thus a key aspect of urban form [34]. Comparing the measures in this study with previous study which focused on an urban setting, it is clear that residential density is considerably low (109.88 vs. 740.3) [35]. In rural settings, it is possible that rather than the simple quantities of housing in neighborhoods, the quality of neighborhoods, manifesting through factors such as social bonding, mutual trust, and prosperity, which are collectively known as social capital, might be more critical for the health of older adults [36]. As rural residents are most likely to move around on their motor vehicles, street connectivity may not be an important factor affecting physical activity and their health, and this finding is consistent with those of previous studies [14]. Parks and recreational center were not associated as they may not be the place for older adults to engage in health-related activities. While this study examined each environmental variable separately, summary index measure is often used in urban neighborhood studies. Walkability index is one of the common indexes which is widely used since it had been developed in 2005 [34]. Although we did not compose such summary index as it was difficult to identify whether each variable have positive or negative effect on weight change based on limited rural studies, it is necessary for future studies to compose index measure that can be generalizable to other rural settings.

One major limitation of this study is that we only analyzed the change in weight, but not in body mass index (BMI) or other more precise body composition measures such as muscle mass. Although our study found an association between land slope and the risk of weight gain, it is controversial whether weight gain among older adults is resulting in an adverse effect on their health. The study conducted a meta-analysis of weight change and all-cause mortality in older adults reported that both weight gain and loss had a higher all-cause mortality risk [pooled Risk Ratio or RR = 1.21, (95% CI 1.09, 1.33); pooled RR 1.67, (95% CI 1.51, 1.85)] [1]. On the other hand, the obesity paradox, which is a phenomenon where BMI-based obesity can be protective or have no effect on specific morbidities or mortality rates from a certain age among older adults has been reported by several population-based cohort studies [37, 38]. Considering the recent evidence of obesity among older adults, future research should use multiple measures, such as weight, BMI, abdominal circumference, and muscle mass, among others.

Another limitation is the relatively small and non-randomized sample of this study. The participants were limited to those who participated in community health checkups and our Shimane CoHRE study. In addition, we were able to follow up only 32% of the subjects invited at the baseline. It might have biased our results, and therefore future studies should consider selecting larger samples at random to reduce potential selection bias. Moreover, although we had information on factors such as the attainment of education, data on other socio-economic status (SES) such as income and occupation were not available. The lack of such data might have overestimated the effect of the neighborhood environment or might have failed to detect its effect across SES groups. In developed countries, it is well confirmed that individual SES, including ethnicity, income and education are associated with health outcomes, such as obesity, diabetes and cardiovascular diseases [39, 40]. In order to achieve equity in health, it is necessary to know whether different SES groups could benefit out from policy or environmental interventions, thus, it is critical to include SES as a potential confounder or effect modifier in the studies investigating the effect of neighborhood environments. While there is no concern for ethnic disparities as Japanese population is homogenous, lack of information of individual income might have biased our results. Nevertheless, government report that higher educational attainment is associated with higher household income consistently in all age groups in Japan [41]. Furthermore, SES is known to be associated with health related behaviors, i.e., smoking and drinking [42, 43], which we could successfully control in our models. We have additionally examined how the effect of neighborhood environment changed before and after including education status in the final models, and found that there was no considerable change of odds of weight gain by slope [400 m buffer without education status: 2nd vs 1st quartile: OR 1.266 (95% CI 0.516, 3.106), 3rd vs 1st: OR 2.234 (95% CI 1.003, 4.974), 4th vs 1st: OR 2.478 (95% CI 1.136, 5.405)]. While these could partially compensate for lack of other SES factors, we were unable to assess whether the final sample differed in SES compared with the original sample, which may have hampered the validity of both the models and the final sample. Besides, lack of controlling comorbidities might have biased our results as they might have been confounding for the association neighborhood environment and weight change.

Finally, available data time of environment variables were not exactly the same as the study period, and all environment variables were measured at one time point. In this study, although there are variables that are less likely to change within 3 years, such as land slope and public transit stops, measurement bias for possible longitudinal change of neighborhood environment cannot be neglected. Not capturing all physical environments that can possibly affect weight change, e.g., food environment, also limited our analysis. As weight change occurs by energy balance, i.e., physical activity (energy expenditure) and diet (energy intake), it is important to take into account both aspects in neighborhood studies. A recent systematic review on the association between neighborhood physical environment and adults’ weight status pointed out that few studies considered individual diet as a mediator [44]. The major reasons why many studies fail to include diet are that difficulty of obtaining accurate individual diet habit information, and mediation analysis which require complex statistics, such as multilevel structural equation modeling. However, it is critical for future studies need to capture temporal as well as longitudinal data for neighborhood environment, as well as taking into account for both physical activity and diet as mediators to elucidate the mechanism of association between neighborhood environment and weight status. Despite these limitations, this study was significant in that it presented new evidence that the neighborhood environment attributes specific to a rural area, i.e., land slope can affect population health outcomes while focusing on longitudinal weight change status among Japanese older adults. Findings from this study intensify the further needs for both rural and region-specific studies and make a case for the accumulation of more evidence to contribute to the development of evidence-based interventions to facilitate healthy aging.

## Conclusion

In conclusion, a hilly neighborhood was found to be an environmental attribute specific to a rural area with the capacity to result in an increased risk of longitudinal weight gain among older adults in Japan. More regional studies should be carried out in this field with a focus on region-specific environmental attributes that can affect the health of older adults.

## Data Availability

The data used in this paper include personal residential information as well as health related information. Therefore the data are not publicly available due to privacy concerns. The data could be requested and available from the corresponding author on safe manner in accordance with the ethical policy statements of this study protocol.
